# The Regionalization of High‐Volume Cardiac Pacing: A Prospective Cohort Study on Safety, Feasibility, and the Learning Curve in a Geriatric Population

**DOI:** 10.1002/clc.70479

**Published:** 2026-09-29

**Authors:** Sergejs Pikta, Deniss Vasiļjevs, Kaspars Kupics, Oskars Kalejs, Nikolajs Nesterovics

**Affiliations:** ^1^ Department of Cardiology and Internal Medicine Daugavpils Regional Hospital Daugavpils Latvia; ^2^ Medical Faculty Riga Stradins University Riga Latvia; ^3^ Latvian Cardiology Centre Pauls Stradins Clinical University Hospital Riga Latvia; ^4^ Faculty of Medicine University of Latvia Riga Latvia

**Keywords:** cardiac pacing, CUSUM analysis, geriatric cardiology, healthcare decentralization, healthcare equity, regional hospital

## Abstract

**Background:**

Centralizing pacemaker implantations delays care for geriatric populations in remote regions, particularly in post‐Soviet healthcare systems like Latvia. We hypothesized that a regional hospital could safely establish a high‐volume pacing program to overcome geographical barriers, utilizing structured mentorship from a national tertiary center.

**Methods:**

This 2‐year prospective observational study evaluated 173 consecutive patients (mean age 78.1 years) undergoing permanent pacemaker implantation at Daugavpils Regional Hospital. Procedures were performed by two regional interventional cardiologists transitioning to independent practice, supported by Latvian Cardiology Centre mentors. Endpoints included 30‐day major complications, procedural efficiency, surgical learning curve evaluation using Cumulative Sum (CUSUM) analysis, and patient‐reported quality of life (AQUAREL, SF‐36).

**Results:**

Across 173 procedures (66.5% dual‐chamber, 33.5% single‐chamber), the overall complication rate was 5.7%. CUSUM analysis demonstrated a complication rate reduction from 8.7% during the initial acquisition phase to a 3.4% expertise plateau. Procedural efficiency stabilized with a mean fluoroscopy time of 3.9 min. Specific complications included lead dislodgement (2.3%), pocket hematoma (1.7%), pneumothorax (1.1%), and wound infection (0.6%), managed successfully without mortality or device abandonment. At 6‐month follow‐up, patients reported significant quality of life improvements and high trust in the regional team.

**Conclusions:**

Regional high‐volume cardiac pacing programs are safe and vital for equitable geriatric care. With structured mentorship, regional hospitals can overcome historical centralization, safely transitioning to independent high‐volume centers and achieving an expert‐level safety profile non‐inferior to tertiary standards.

## Graphical abstract

This prospective study demonstrates that regionalized high‐volume cardiac pacing programmes, supported by structured mentorship, are safe and efficient. Regional centres can successfully navigate the surgical learning curve, achieving safety outcomes non‐inferior to tertiary standards while eliminating geographical barriers for elderly patients.

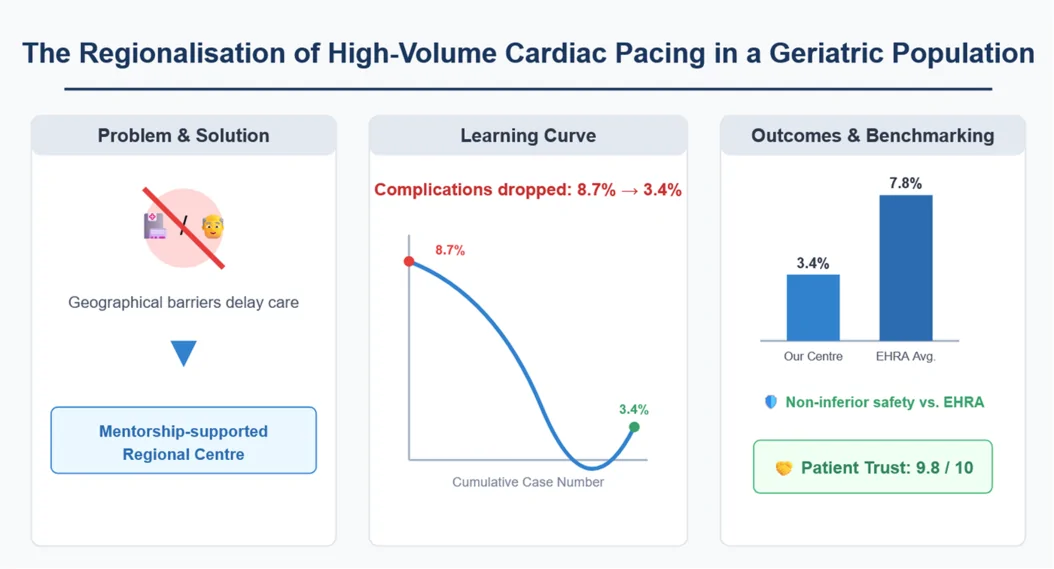

## 1 Introduction

The burden of symptomatic bradyarrhythmias is increasing across Europe, driven primarily by an ageing population. While the European Society of Cardiology (ESC) provides clear clinical guidelines for the management of these conditions [1], the equitable implementation of these services in rural and regional areas remains a profound logistical and policy challenge.

Current healthcare paradigms strongly favor the centralization of device implantations in tertiary, university‐affiliated hospitals [2]. This model is predicated on the assumption that high procedural volumes in centralized locations are the sole means of ensuring patient safety and minimizing complications [3]. However, for geriatric populations living in remote regions, this creates a severe “geographical barrier” [4]. Forced centralization often leads to delayed treatments, logistical hardships for patients and their families, and substantial psychological stress, highlighting a critical gap in healthcare equity.

In post‐Soviet healthcare systems, such as in Latvia, cardiac care has traditionally followed a highly centralized model. Historically, advanced electrophysiology and permanent pacemaker (PPM) implantations have been strictly restricted to university centers in Riga, the national capital. For citizens residing in Eastern Latvia (Latgale), accessing these life‐saving procedures required traversing geographical distances of up to 230 km. In real‐world clinical practice, emergency or urgent inter‐hospital transfer of hemodynamically unstable geriatric patients over such distances is fraught with logistical hurdles, frequently resulting in transfer delays ranging from 6 to 24 h depending on ambulance availability and tertiary intensive care unit bed capacity. Furthermore, patients presenting locally during overnight hours or weekends with life‐threatening high‐grade atrioventricular block historically required maintenance on temporary transvenous pacing catheters in regional intensive care units while awaiting transfer—a practice associated with recognized risks of catheter dislodgement, venous thrombosis, and secondary sepsis.

This centralization conservatism was driven by administrative tradition rather than technical limitations, creating significant “distance decay” in healthcare quality and accessibility. There is a critical need to evaluate whether transitioning to a decentralized, regionalized model can maintain surgical safety while improving patient outcomes.

We hypothesized that a regional hospital could bypass the limitations of strict centralization and provide “Centre of Excellence” quality care locally. By implementing a structured mentorship programme with a national tertiary centre (Pauls Stradins Clinical University Hospital, Latvian Cardiology Centre), we aimed to evaluate the clinical feasibility, safety, and learning curve associated with establishing a high‐volume cardiac pacing programme in a regional geriatric population. Ultimately, decentralized, high‐volume pacing programmes provide a scalable, evidence‐based alternative to strict centralization, effectively eliminating access inequality and patient travel burdens without compromising clinical outcomes.

## 2 Methods

### 2.1 Study Design, Healthcare Setting, and Patient Consent

This was a prospective observational cohort study conducted at Daugavpils Regional Hospital (Daugavpils, Latvia) over a 2‐year period (2024–2025). Daugavpils Regional Hospital is the primary multi‐profile medical facility serving the Latgale region in southeastern Latvia, supporting a regional catchment area of approximately 250,000 inhabitants located up to 250 km from the national tertiary centers in Riga. To address geographical health inequities and eliminate inter‐hospital transfer delays, we implemented a decentralized Hub‐and‐Spoke healthcare delivery model. In this framework, our regional hospital (the ‘Spoke') transitioned from a non‐implanting referring facility into an independent high‐volume pacing center, supported by formal procedural mentorship from the national tertiary center (the ‘Hub').

We enrolled 173 consecutive patients who presented with symptomatic bradycardia meeting standard ESC criteria for permanent pacing [1]. There were no clinical exclusion criteria, ensuring the cohort represented a true “real‐world” demographic. The study protocol was approved by the Institutional Ethics Committee. Crucially, while all pacemaker implantations adhered strictly to standard‐of‐care clinical guidelines and routine surgical techniques without experimental device modification, all participants provided written informed research consent prior to inclusion. This research consent specifically governed: (1) prospective enrollment into the analytical institutional database; (2) systematic 30‐day clinical complication tracking; and (3) the administration of validated quality‐of‐life and satisfaction survey instruments at baseline and 6‐month follow‐up. The research was conducted in full accordance with the Declaration of Helsinki.

### 2.2 Operator Details and Mentorship Structure

A fundamental distinction of our regionalization model relates to operator workforce structure. In contrast to healthcare systems (such as the UK National Health Service) where electrophysiologists routinely operate across both district general hospitals and tertiary centers within a split weekly job plan, regional operators in Latvia are permanently dedicated to their regional institution. In this study, procedures were performed by two primary local interventional cardiologists based full‐time at Daugavpils Regional Hospital. Both operators had completed formal fellowship training in interventional cardiology and basic device management at university clinics in Riga, supplemented by specialized observational clinical training courses at high‐volume European electrophysiology centers (including procedural training at Policlinico Universitario Agostino Gemelli, Rome, Italy). Prior to program initiation, both operators experienced high annual volumes in percutaneous coronary interventions (PCI) and temporary pacemaker placements, but possessed limited independent primary volume for permanent device implantation.

Crucially, the programme was structured around a mentorship‐based transition model designed to achieve high‐volume competency locally without cross‐site commuting. During Phase 1 (Acquisition, Cases 1–57), initial implantations were performed under the direct on‐site proctorship of senior visiting electrophysiology supervisors from the Latvian Cardiology Centre who assisted in the sterile field. Once local operator proficiency and electrical parameter stabilization were confirmed, proctorship transitioned to Phase 2 (Stabilization, Cases 58–115) and Phase 3 (Expertise, Cases 116–173), consisting of remote pre‐procedural anatomical review and retrospective quality audits. By concentrating the regional pacing caseload between two dedicated local physicians, each operator achieved an annual procedural volume exceeding 75–80 cases/year locally, classifying them as medium‐to‐high volume operators by European Heart Rhythm Association (EHRA) standards.

### 2.3 Catheterization Laboratory Setup and Support Staff

To evaluate the feasibility of regional decentralization, we compared our local technical infrastructure against standard tertiary environments. The catheterization laboratory at Daugavpils Regional Hospital operates 24/7 for acute cardiovascular emergencies and is physically equipped with state‐of‐the‐art angiographic fluoroscopy C‐arms, multi‐channel invasive hemodynamic monitoring systems, integrated electrophysiological recording displays, and advanced resuscitation equipment, rendering it functionally identical to university tertiary cath lab suites.

The lab support staff comprises specialized cardiovascular nurses and certified radiographers with extensive daily expertise in sterile interventional field management, endovascular instrumentation, and emergency pharmacotherapy. Prior to program launch, the nursing and technical staff underwent targeted didactic and practical training proctored by visiting tertiary electrophysiology nurses, focusing on pacing generator preparation, sterile lead handling, analyzer threshold testing protocols, and pocket hemostasis assistance. All implantations were performed using a standardized surgical protocol via subclavian vein puncture or baseline cephalic vein cutdown. Depending on the clinical indication, patients received either dual‐chamber (DDD/DDDR) or single‐chamber (VVI/VVIR) systems.

### 2.4 Outcomes and Statistical Analysis

The primary endpoint was clinical safety, defined as the incidence of 30‐day major procedure‐related complications (including lead dislocation, systemic infection, pneumothorax, clinically significant hematoma requiring intervention, or procedure‐related mortality). Secondary endpoints included procedural efficiency (measured by total procedure time and fluoroscopy time) and changes in patient‐reported quality of life (QoL). QoL and symptom burden were assessed pre‐operatively and at 6 months post‐operatively using the validated AQUAREL (pacemaker‐specific) [5] and SF‐36 (general health) [6] questionnaires. Subjective patient satisfaction and trust were assessed using a 10‐point Visual Analogue Scale (VAS).

To evaluate the evolution of procedural safety and efficiency, a Cumulative Sum (CUSUM) analysis was employed to map the surgical learning curve across the 173 consecutive procedures [7]. Continuous variables were expressed as mean ± standard deviation (SD). Pre‐ and post‐operative QoL scores were compared using a paired Student's *t*‐test. A *P*‐value of < 0.05 was considered statistically significant. All statistical analyzes were performed using IBM SPSS Statistics for Windows, Version 29.0 (IBM Corp., Armonk, NY, USA).

## 3 Results

### 3.1 Baseline Clinical and Procedural Characteristics

The baseline demographic, clinical, and intraoperative characteristics of the study cohort are summarized in Table 1. The study population reflects a typical real‐world geriatric demographic, with a mean age of 78.1 ± 9.2 years (range: 48–97 years) and a slight female predominance (54.3%). The primary clinical indications for permanent pacemaker implantation were high‐grade atrioventricular (AV) block, which accounted for 61.8% of cases, followed by sick sinus syndrome (SSS) in 38.2%.

**Table 1:** Baseline clinical characteristics and intraoperative electrical parameters of the study cohort (*n* = 173).

Characteristic	Value (Mean ± SD or n, %)
**Demographics**	
Age, years (Mean ± SD)	78.1 ± 9.2
Age range, years	48–97
Gender (Female/Male)	94 (54.3%)/79 (45.7%)
**Clinical indications**	
AV Block (II–III degree)	107 (61.8%)
Sick Sinus Syndrome (SSS)	66 (38.2%)
**Procedure details**	
Dual‐Chamber (DDD/DDDR)	115 (66.5%)
Single‐Chamber (VVI/VVIR)	58 (33.5%)
Elective device replacement	25 (14.5%)
First‐time implantation	148 (85.5%)
**Intraoperative electrical parameters**	
Ventricular Lead (RV)—*R*‐wave Sensing (mV)	11.4 ± 3.8
Ventricular Lead (RV)—Pacing Threshold (V at 0.4 ms)	0.75 ± 0.21
Ventricular Lead (RV)—Impedance (Ω)	548 ± 112
Atrial Lead (RA, DDD cases only)—*P*‐wave Sensing (mV)	2.8 ± 1.1
Atrial Lead (RA, DDD cases only)—Pacing Threshold (V at 0.4 ms)	0.82 ± 0.28
Atrial Lead (RA, DDD cases only)—Impedance (Ω)	512 ± 95
**Efficiency metrics**	
Mean procedure time (min)	78.4 ± 14.2
Mean fluoroscopy time (min)	3.9 ± 1.1

The vast majority of the cohort (85.5%) underwent first‐time (de novo) device implantation, while 14.5% underwent elective generator replacement. Dual‐chamber pacing systems (DDD/DDDR) were implanted in 66.5% of patients, whereas 33.5% received single‐chamber (VVI/VVIR) devices. Intraoperative testing demonstrated excellent electrical stability and optimal lead performance across all procedures achieving R‐wave sensing and pacing thresholds fully non‐inferior to European standards (Figure 3C), (Table 1).

### 3.2 Procedural Efficiency and Learning Curve

Analysis of procedural times revealed a distinct three‐phase evolution (Figure 1):

- **Acquisition (Cases 1–57):** Mean procedure time was 120 min.
- **Stabilization (Cases 58–115):** Mean procedure time decreased to 85 min.
- **Expertise (Cases 116–173):** Mean procedure time plateaued at 78 min.

**Figure 1:**
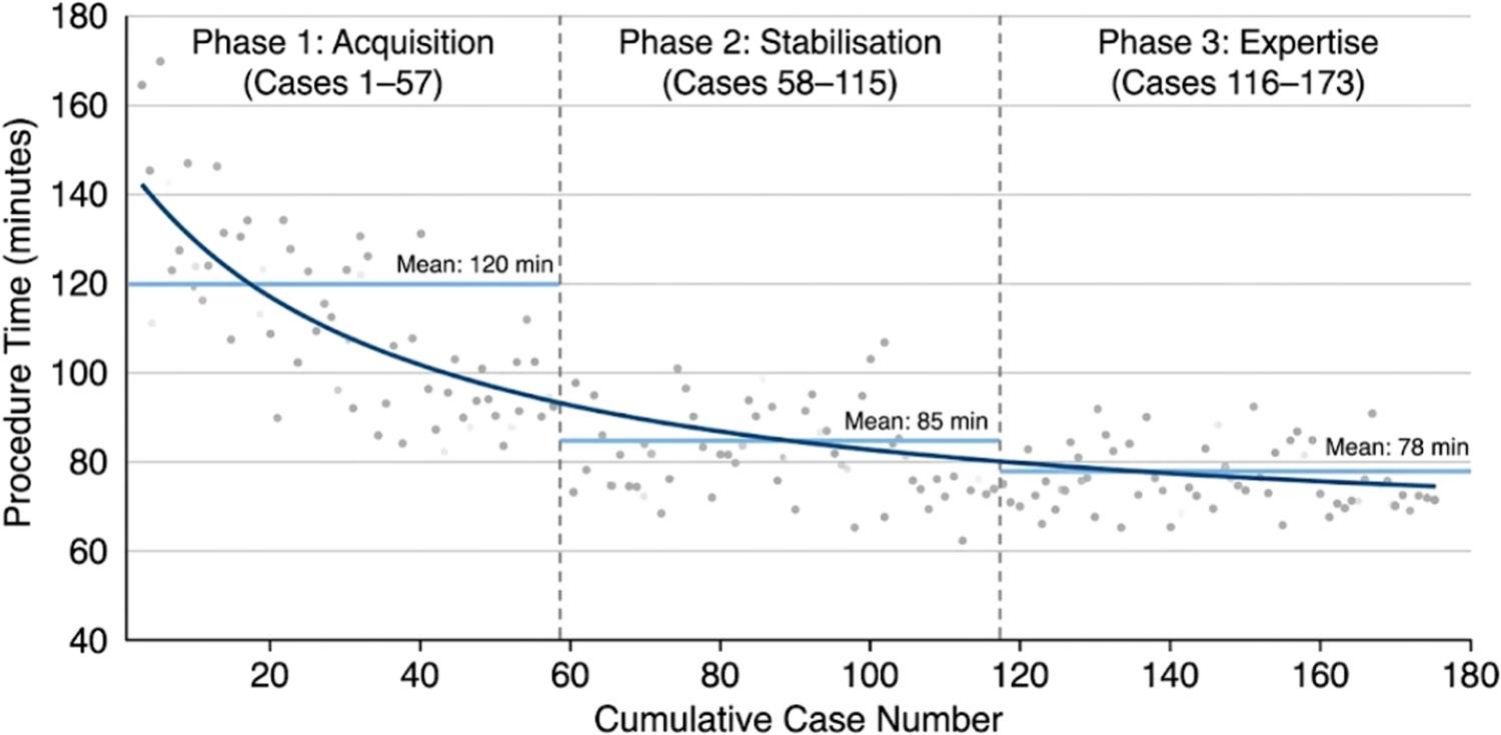
Evolution of procedural efficiency across the surgical learning curve. The graph demonstrates the relationship between cumulative case volume (*x*‐axis) and total procedure time in minutes (*y*‐axis). The overall learning curve is demarcated into three distinct clinical phases: the initial Acquisition phase (Cases 1–57; mean time 120 min), the Stabilization phase (Cases 58–115; mean time 85 min), and the final Expertise phase (Cases 116–173; mean time 78 min). The trendline illustrates a continuous reduction in procedural duration, highlighting that optimal surgical efficiency is consistently achieved and stabilized after approximately 100 cases. Figure 1: Learning Curve (Time vs. Case Number).

By the final phase, the regional programme achieved high technical efficiency and radiation safety, characterized by a mean total procedure time of 78.4 ± 14.2 min and an exceptionally low mean fluoroscopy exposure time of 3.9 ± 1.1 min (Figure 3B).

### 3.3 Safety and Complications (CUSUM Analysis)

The overall total complication rate across the entire cohort was 5.7% (10 out of 173 patients). Lead dislocations occurred in 2.3% of cases, predominantly within the first 60 cases. There was no procedure‐related mortality or deep systemic/pocket infection.

The CUSUM Safety Learning Curve (Figure 2) demonstrated a significant shift across the three phases. In Phase 1 (Acquisition), the complication rate was 8.7%, primarily associated with prolonged venous access and early technical adjustments. In Phase 2 (Stabilization), safety improved markedly, with complications dropping to 5.2% as surgical routines standardized. In Phase 3 (Expert Plateau), the complication rate reached an expert‐level plateau of 3.4%. Complications in this final phase were strictly associated with complex patient anatomy (e.g., severely dilated right atria) rather than technical errors.

**Figure 2:**
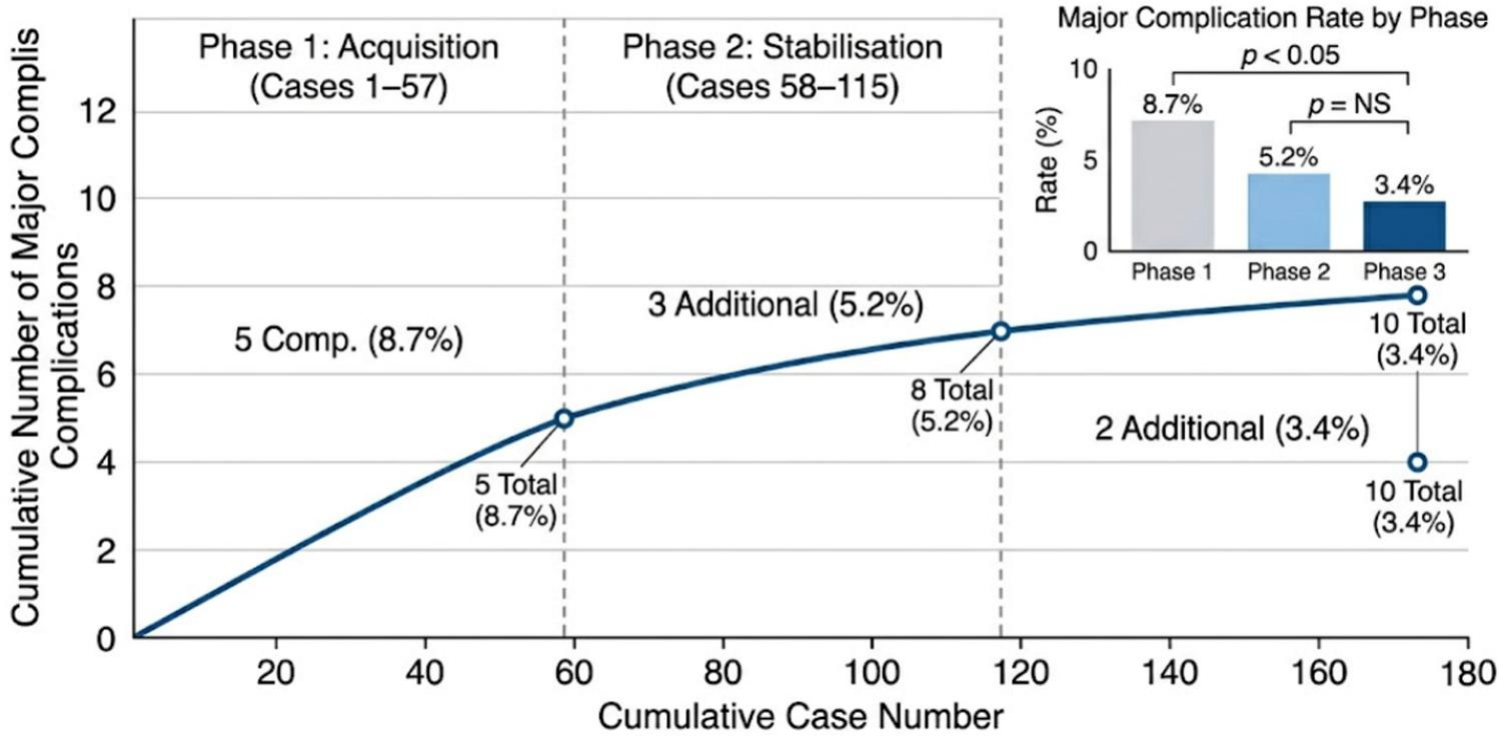
Cumulative Sum (CUSUM) safety analysis of major procedure‐related complications. This CUSUM curve tracks the cumulative incidence of major complications across the 173 consecutive pacemaker implantations. The slope of the curve represents the complication rate at a given stage of the programme's maturity. A steeper slope is observed during Phase 1 (Acquisition, 8.7% rate), reflecting early procedural adjustments. The curve visibly flattens through Phase 2 (Stabilization, 5.2% rate) and reaches a sustained safety plateau in Phase 3 (Expertise), where the major complication rate drops to 3.4%. The embedded bar chart highlights the statistically significant reduction in major complications between the initial and expert phases (*p* < 0.05). Figure 2: CUSUM Safety Curve (Complications vs. Case Number).

### 3.4 Detailed Complication Analysis and Procedural Learning Points

To provide complete clinical transparency and outline the practical drivers of our learning curve, Table 2 itemizes all observed major and minor complications, their management protocols, and the resulting clinical adaptations:

- *Lead Dislodgement (n = 4, 2.3%)*: All instances involved right ventricular lead micro‐ or macro‐dislocation occurring within 48 h post‐implantation (three in Phase 1, one in Phase 2). All cases were successfully managed via percutaneous transvenous lead repositioning and securement in the catheterization laboratory without lasting sequelae. *Learning Point:* Analysis of early dislodgements prompted a mandatory procedural protocol change: operators instituted routine intraoperative threshold and sensing validation under deep‐breathing and forced coughing maneuvers, alongside leaving an optimized, unstrained atrial loop to accommodate diaphragmatic excursion.
- *Pocket Hematoma (n = 3, 1.7%)*: Clinically significant pocket hematomas occurred in three patients (two in Phase 1, one in Phase 2). Two patients were managed conservatively with continuous pressure dressings and temporary antiplatelet withholding, achieving complete resorption. One patient on uninterrupted direct oral anticoagulants (DOACs) combined with aspirin required surgical pocket exploration, hematoma evacuation, and electrocautery on post‐operative day 3, with secondary closure. No hematoma progressed to infection. *Learning Point:* Re‐emphasized the necessity of meticulous sub‐cuticular hemostasis and accelerated the standardized adoption of ESC guidelines for perioperative DOAC bridging/timing schedules, utilizing bipolar electrocautery for all muscular pocket beds.
- *Pneumothorax (n = 2, 1.1%)*: Two patients developed localized pneumothoraces during Phase 1 following anatomical subclavian vein puncture attempts. One localized apical pneumothorax resolved spontaneously with conservative observation and oxygen supplementation. One patient required a temporary 24‐h small‐bore pleural pigtail catheter insertion, achieving full lung re‐expansion. *Learning Point:* This complication directly drove a technical evolution in our venous access strategy. The regional team transitioned from blind anatomical subclavian puncture to baseline cephalic vein cutdown as the primary preferred access route, utilizing real‐time ultrasound‐guided axillary/subclavian puncture as the mandatory secondary approach. This adaptation completely eliminated pneumothorax events during Phase 2 and Phase 3.
- *Superficial Wound Infection (n = 1, 0.6%)*: One patient presented at post‐operative Day 14 with localized incisional erythema without deep pocket involvement. Successfully treated with a 10‐day outpatient course of oral amoxicillin/clavulanic acid based on sensitivity profiling, preserving the device. *Learning Point:* Reinforced strict adherence to pre‐operative intravenous antibiotic prophylaxis timing (cefazolin administration 30–60 min prior to skin incision).

### 3.5 Patient‐Reported Outcomes and Quality of Life

Patient‐reported outcomes and health‐related QoL at the 6‐month follow‐up demonstrated highly significant improvements across all measured metrics (Table 3).

**Table 2:** Summary of major complications, clinical management, and procedural learning points (*n* = 173).

Complication type	Incidence (*n*, %)	Learning curve phase	Clinical management & outcome	Procedural learning point & adaptation
Lead dislodgement (RV)	4 (2.3%)	Phase 1 (*n* = 3) Phase 2 (*n* = 1)	Percutaneous transvenous lead repositioning in cath lab under fluoroscopy. Successful capture restored in all 4 cases without permanent sequelae.	Transition from stiff interventional wires to flexible pacing leads required tactile refinement. *Action:* Instituted mandatory intraoperative threshold testing under forced breathing/coughing maneuvers and left a generous, unstrained atrial loop.
Pocket hematoma	3 (1.7%)	Phase 1 (*n* = 2) Phase 2 (*n* = 1)	Two patients managed conservatively with compression dressings. One patient on uninterrupted DOAC required surgical pocket exploration and electrocautery evacuation on POD 3. No secondary infections.	Highlighted the need for meticulous sub‐cuticular hemostasis. *Action:* Adopted strict adherence to ESC guidelines on perioperative DOAC timing/bridging schedules and mandated routine bipolar electrocautery for all muscular pocket beds.
Pneumothorax	2 (1.1%)	Phase 1 (*n* = 2) Phase 2 (*n* = 0) Phase 3 (*n* = 0)	One apical pneumothorax resolved spontaneously with conservative observation and supplemental oxygen. One required temporary (24 h) small‐bore pleural pigtail catheter drainage with complete re‐expansion.	Anatomical blind subclavian puncture carried unacceptable pleural risk during early volume acquisition. *Action:* Switched primary preferred venous access route to baseline cephalic vein cutdown, utilizing ultrasound‐guided axillary puncture as secondary backup. Zero events in Phase 2/3.
Superficial wound infection	1 (0.6%)	Phase 2 (*n* = 1)	Incisional erythema at POD 14 treated successfully with a 10‐day outpatient course of oral amoxicillin/clavulanic acid based on swab culture. No deep pocket involvement; device preserved.	Reinforced strict procedural asepsis. *Action:* Standardized mandatory pre‐operative intravenous antibiotic prophylaxis timing (cefazolin infusion completed 30–60 min prior to skin incision).

**Table 3:** Patient‐reported outcomes and quality of life assessment (*n* = 173).

Domain/Metric	Pre‐operative (Baseline)	Post‐operative (6 Months)	*p*‐value
**AQUAREL score (Pacemaker‐specific)**			
— Chest pain/Discomfort	42.5 ± 12.1	18.2 ± 6.4	< 0.001
— Arrhythmia symptoms	58.1 ± 15.4	12.5 ± 5.2	< 0.001
— Exertional Dyspnea (Shortness of breath)	64.3 ± 18.2	24.1 ± 8.7	< 0.001
**SF‐36 Health Survey Domains**			
— Physical functioning	38.4 ± 10.5	62.7 ± 12.8	< 0.001
— Vitality/Energy levels	31.2 ± 9.8	58.4 ± 11.2	< 0.001
— Mental Health/Emotional well‐being	45.6 ± 11.4	71.2 ± 10.5	< 0.001
**Subjective satisfaction (VAS 1–10)**			
— Trust in regional medical Team	N/A	9.8 ± 0.4	—
— Ease of access (Proximity to home)	N/A	9.7 ± 0.5	—
— Overall procedure satisfaction	N/A	9.9 ± 0.2	—

Evaluation of pacemaker‐specific symptom burden (AQUAREL) revealed a marked reduction in clinical symptoms. Mean scores for chest pain and discomfort decreased from 42.5 ± 12.1 at baseline to 18.2 ± 6.4 at 6 months. Patients also reported significant relief from arrhythmia symptoms and a pronounced reduction in exertional dyspnoea. General QoL (SF‐36) showed corresponding enhancements, with Physical Functioning, Vitality, and Mental Health domains all improving significantly.

Subjective patient satisfaction (VAS) was exceptionally high at 6 months. Patients reported an overall satisfaction score for the procedure of 9.9 ± 0.2. The cohort demonstrated profound trust in the regional medical team (9.8 ± 0.4) and high satisfaction regarding the ease of access and proximity to care (9.7 ± 0.5).

## 4 Discussion

### 4.1 Summary of Main Findings

Our study demonstrates that establishing a high‐volume regional pacing programme is not only clinically feasible but highly safe. Through a structured mentorship approach, our regional centre successfully navigated the surgical learning curve, reducing initial complication rates from 8.7% to a stabilized expert plateau of just 3.4% after roughly 100 cases. Concurrently, the programme delivered profound improvements in patient quality of life and generated immense trust within the local community.

Our findings dismantle the “centralization myth” by proving that high‐volume regional centers can achieve safety plateaus (3.4% major complication rate) non‐inferior to international benchmarks. The implementation of the Hub‐and‐Spoke model effectively eliminated geographical barriers for a fragile geriatric population. The significant improvement in patient‐reported quality of life (AQUAREL and SF‐36 scores) and the high level of trust (9.8/10) demonstrate that local accessibility is a key determinant of success in modern cardiac rhythm management.

### 4.2 Comparison With Other Studies

The data decisively challenge the “low‐volume, high‐risk” trap often associated with regional hospitals. By concentrating local pathology between two full‐time regional interventional cardiologists, we achieved an expertise phase complication rate (3.4%) that is significantly lower than the EHRA average of 7.8% reported by larger, established centres (Figure 3A) [2, 8]. Furthermore, our stabilized mean fluoroscopy time of 3.9 min places our regional centre in the highest‐performing quartile of Western European benchmarks, underscoring strict adherence to radiation safety protocols.

**Figure 3:**
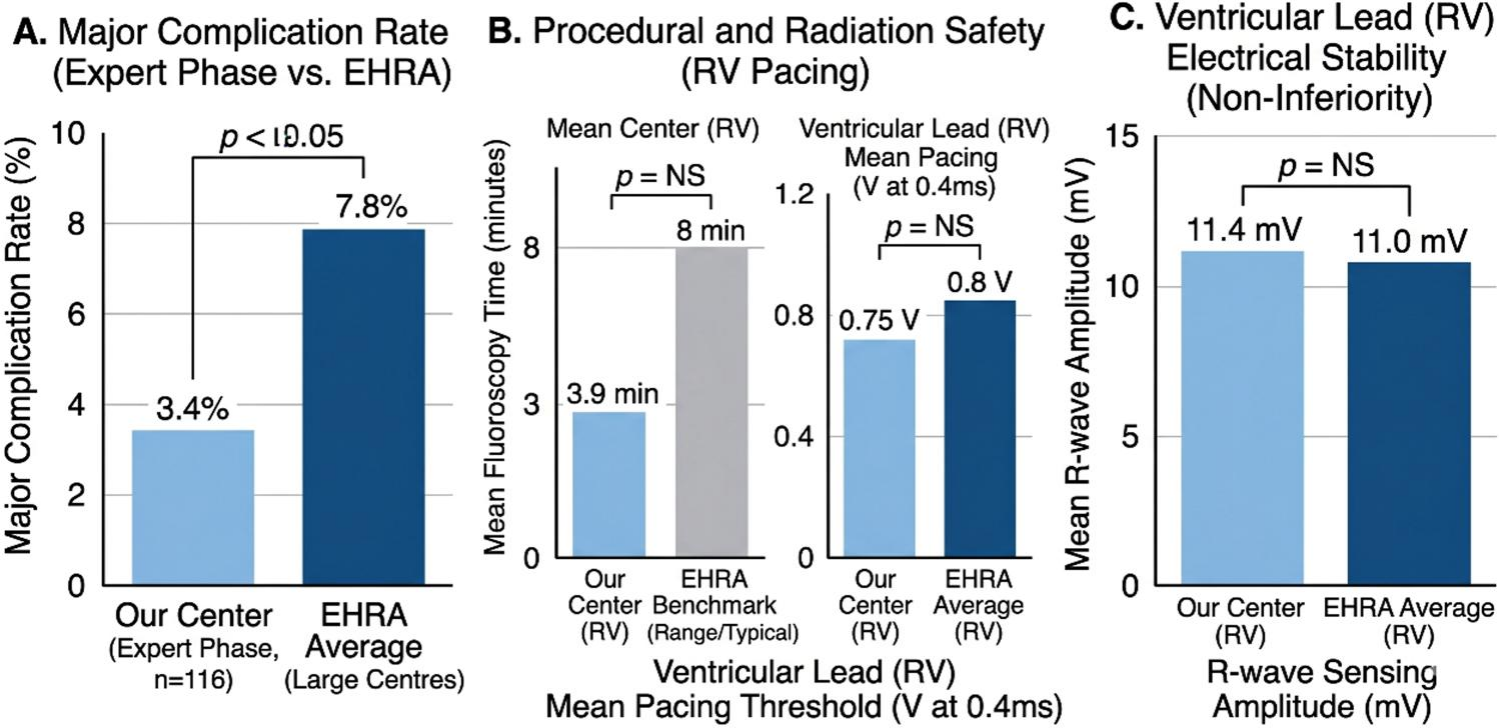
Benchmarking of clinical safety and procedural outcomes against European Heart Rhythm Association (EHRA) averages. (A) The major complication rate of the regional centre during its mature Expertise Phase (*n* = 116) compared to the established EHRA registry average. The regional programme demonstrated a significantly lower complication rate (3.4% vs. 7.8%; *p* < 0.05). (B) Procedural and radiation safety metrics, illustrating the centre's exceptionally low mean fluoroscopy time (3.9 min) compared to typical Western European benchmarks. (C) Intraoperative electrical stability (mean R‐wave sensing amplitude and ventricular pacing threshold), confirming that the regional centre achieves optimal, non‐inferior lead performance parameters. Figure 3: Benchmarking Bar Chart (Our Centre vs. EHRA Averages).

### 4.3 Challenging Historical Conservatism and Service Scope

A primary contribution of this study lies in its direct challenge to the historical conservatism prevalent in post‐Soviet and developing healthcare systems. While district general hospitals in the UK and Western Europe routinely perform a wide array of electrophysiological interventions—including conduction system pacing (CSP), implantable cardioverter‐defibrillators (ICD), and cardiac resynchronization therapy (CRT)—regional facilities in Latvia were historically restricted to diagnostic or temporary procedures due to centralized administrative dogma rather than physical cath lab limitations.

Specifically, prior to 2023, the implantation of all permanent cardiac rhythm devices—including standard pacemakers (PPM), ICDs, and CRT systems—was strictly limited to only two university tertiary centers located in the capital city of Riga. In 2023, two dedicated regional interventional cardiologists at Daugavpils Regional Hospital recognized the profound human and systemic cost of this centralization. Driven by the motivation to improve patient care and eliminate the physical, emotional, and financial burdens forced upon geriatric patients and their families traveling across the country, these operators initiated the regional pacing program. This initiative was equally motivated by the desire to alleviate the significant logistical strain on local emergency medical services and regional nursing staff, who were previously burdened with organizing, accompanying, and managing long‐distance inter‐hospital ambulance transfers for pacemaker‐dependent patients.

By establishing a rigorous, proctored Hub‐and‐Spoke model and documenting an expert‐phase safety profile non‐inferior to European registries, we have proven that regional catheterization laboratories equipped with modern imaging and experienced interventional personnel can independently execute permanent pacemaker implantations. While our initial 2‐year protocol intentionally restricted the procedural scope to standard single‐ and dual‐chamber pacing (VVI/DDD) to establish a baseline foundation of surgical excellence without diluting operator volumes, this program proves that strict centralization is no longer mandatory. The successful mastery of basic pacing serves as the essential institutional prerequisite for the subsequent safe regional introduction of complex device therapies (ICD, CRT, and CSP) in the near future.

### 4.4 Clinical and Policy Implications

For an ageing demographic (mean age 78.1 in our cohort), the logistical and psychological burden of travelling to a distant tertiary centre is substantial [4]. Eliminating the 230 km travel barrier and avoiding prolonged overnight stays on temporary pacing in regional ICUs directly mitigates procedural morbidity. The exceptionally high trust score (9.8/10) reported in our study strongly suggests that receiving advanced procedural care “close to home” by a familiar medical team significantly reduces perioperative anxiety. Furthermore, the inclusion of 14.5% elective replacements indicates that our regional centre has matured into a full‐cycle facility, capable of safely managing long‐term device replacements [9]. This decentralized model offers a sustainable solution to regional health disparities.

To facilitate the replication of this regionalization model in similar community hospitals, several practical logistical factors must be considered. First, the successful initiation of such a program relies heavily on the clinical enthusiasm and dedication of the local medical team, as the training phase was undertaken without additional financial remuneration for the physicians. The proctorship arrangement was made possible through educational support from device manufacturers, who sponsored the visiting expert mentors. Regarding support staff, experienced nurses from the local cardiology department and catheterization laboratory were selected for the project. Their initial upskilling was funded by the local hospital as formal professional development, which included training rotations at the tertiary university hospital, followed by hands‐on, on‐site teaching by the visiting proctors. Notably, the specific professional role of a “cardiac physiologist” does not exist in our national healthcare system; therefore, all device programming and physiological assessments are performed directly by the implanting cardiologist.

While formal cost‐effectiveness calculations are beyond the scope of this study, the economic and logistical benefits of establishing this local service are profound, particularly for acute cases. Currently, a patient presenting with complete heart block can be admitted, operated on the following day, and safely discharged shortly thereafter. This paradigm shift drastically reduces local intensive care unit (ICU) bed occupancy and completely eliminates the logistical burden and high costs associated with inter‐hospital transfers via specialized intensive care ambulance teams to the tertiary centre.

### 4.5 Strengths and Limitations

The primary strength of this study is its prospective, consecutive “real‐world” design, which eliminates selection bias. The use of a transparent CUSUM analysis provides a mathematically rigorous mapping of the surgical learning curve [7], offering a precise benchmark (100 cases) for other regional hospitals aiming to adopt this model. Limitations include the single‐centre nature of the study and a cohort size that, while substantial for a regional Baltic centre, is relatively small compared to multinational registries. Additionally, the study lacks a direct, randomized control group within the same institution; however, this was mitigated by benchmarking our outcomes against established EHRA registry data [8].

## 5 Conclusions

Establishing a high‐volume regional pacing programme is a safe, efficient, and highly necessary strategy for modern European cardiology. Our findings dismantle the traditional necessity of strict centralization, proving that with appropriate mentorship and dedicated local operator volume, regional centres can safely eliminate geographical barriers, achieve outcomes non‐inferior to Western European tertiary standards, overcome historical service conservatism, and foster unparalleled patient trust.

## Author Contributions

S.P. conceptualized the study and designed the prospective protocol. S.P., K.K. and N.N. performed the clinical procedures and were responsible for procedural safety and clinical supervision. D.V. was responsible for data collection, patient follow‐up, and the administration of quality‐of‐life questionnaires. D.V. performed the statistical and CUSUM analyzes. S.P. drafted the initial manuscript. K.K., N.N. and O.K. provided expert mentorship and critically revised the manuscript for important intellectual content. All authors had full access to all the data in the study and accept responsibility for the decision to submit for publication.

## Funding

The authors have nothing to report.

## Conflicts of Interest

The authors declare no conflicts of interest.

## Supporting information


Supporting File

## Data Availability

De‐identified individual participant data, along with the study protocol and statistical analysis plan, will be made available upon reasonable request to the corresponding author, following publication, for researchers whose proposed use of the data has been approved by an independent review committee.
