# Supplementary material for: The Regionalization of High‐Volume Cardiac Pacing: A Prospective Cohort Study on Safety, Feasibility, and the Learning Curve in a Geriatric Population
Source: Clin Cardiol. 2026 Sep 29;49(10):e70479. doi: 10.1002/clc.70479 (PMC13621928; doi:10.1002/clc.70479)
Supplement: Supplementary file 1 [file CLC-49-e70479-s001.pdf]

Journal of Cardiovascular Electrophysiology

Original Article

The Regionalisation of High-Volume Cardiac Pacing: A Prospective Cohort Study on Safety, Feasibility, and the Learning Curve in a Geriatric Population

Submission ID d61752d2-97e1-401a-a93a-d256d6010aca

Submission Version Initial Submission

PDF Generation 15 Jun 2026 13:55:20 EST by Atypon ReX

Authors

Dr. Sergejs Pikta, MD
Corresponding Author
Submitting Author

ORCiD
https://orcid.org/0000-0001-7141-4882

Affiliations
- Department of Cardiology and Internal Medicine, Daugavpils Regional Hospital, Daugavpils, Latvia

Dr. Deniss Vasiļjevs, MD

Affiliations
- Department of Cardiology and Internal Medicine, Daugavpils Regional Hospital, Daugavpils, Latvia
- Riga Stradins University, Medical Faculty. Riga, Latvia

Dr. Kaspars Kupics, MD

Affiliations
- Latvian Cardiology Centre, Pauls Stradins Clinical University Hospital, Latvia
- Latvian University, Riga, Latvia

Prof. Oskars Kalejs, MD, PhD

Affiliations
- Latvian Cardiology Centre, Pauls Stradins Clinical University Hospital, Latvia
- Riga Stradins University, Medical Faculty. Riga, Latvia

Prof. Nikolajs Nesterovics, MD, PhD

Affiliations

- Latvian Cardiology Centre, Pauls Stradins Clinical University Hospital, Latvia
- Riga Stradins University, Medical Faculty. Riga, Latvia

Files for peer review

All files submitted by the author for peer review are listed below. Files that could not be converted to PDF are indicated; reviewers are able to access them online.

Name	Type of File	Size	Page
JCE pikta et all manuscr.docx	Main Document - MS Word	1.5 MB	Page 4
figure1.jpg	Figure	106.9 KB	Page 16
figure2.jpg	Figure	124.4 KB	Page 17
figure3.jpg	Figure	185.8 KB	Page 18
Table 1_Baseline Clinical Characteristics.docx	Table	15.3 KB	Page 19
Table_2_Patient-Reported Outcomes and QoL.docx	Table	15.1 KB	Page 21
abstract.png	Graphical Abstract Image	99.2 KB	Page 22

1. Title Page

The Regionalisation of High-Volume Cardiac Pacing: A Prospective Cohort Study on Safety, Feasibility, and the Learning Curve in a Geriatric Population

Authors & Affiliations: Sergejs Pikta, MD[1] , Deniss Vasiļjevs, MD[1,3], Kaspars Kupics, MD[2,4], Oskars Kalejs, MD, PhD[2,3], Nikolajs Nesterovics, MD, PhD [2,3]

[1]Department of Cardiology and Internal Medicine, Daugavpils Regional Hospital, Daugavpils, Latvia

[2] Latvian Cardiology Centre, Pauls Stradins Clinical University Hospital, Latvia

[3] Riga Stradins University, Medical Faculty. Riga, Latvia

[4] Latvian University, Riga, Latvia

Corresponding Author: Sergejs Pikta, MD Department of Cardiology, Daugavpils Regional Hospital, Vasarnicu iela 20, Daugavpils, LV5401, Latvia, Email: sergejs.pikta@gmail.com ORCID: 0000-0001-7141-4882

Keywords: Cardiac pacing • Regional hospital • CUSUM analysis • Geriatric cardiology • Healthcare decentralisation

---

2. Abstract

Aims We aimed to evaluate the feasibility and safety of decentralising cardiac pacing services by establishing a high-volume regional programme to address the geographical barriers faced by geriatric populations.

Methods and results This 2-year prospective cohort study evaluated 173 consecutive patients (mean age 78.1 years) undergoing permanent pacemaker implantation at a regional hospital, supported by structured mentorship from a national tertiary centre. The primary endpoint was the incidence of 30-day major complications, with secondary endpoints including procedural efficiency, patient-reported quality of life (AQUAREL and SF-36), and evaluation of the surgical learning curve using Cumulative Sum (CUSUM) analysis. Over 173 procedures (66.5% dual-chamber, 33.5% single-chamber), the overall complication rate was 5.7%. CUSUM analysis demonstrated a drop in complications from 8.7% during the initial acquisition phase to an expertise plateau of 3.4%. Procedural efficiency was high, with mean fluoroscopy time stabilising at 3.9 minutes. At 6-month follow-up, patients reported highly significant improvements in quality of life and profound trust in the regional medical team (9.8/10).

Conclusion Regional high-volume cardiac pacing programmes are safe, efficient, and vital for equitable geriatric care. With structured mentorship, regional hospitals can overcome the "centralisation myth" and achieve safety outcomes non-inferior to Western European tertiary standards.

Funding: None.

Key messages

- Current healthcare models heavily emphasise centralising cardiac electrophysiological procedures in tertiary hospitals, which inadvertently creates severe geographical barriers and delays care for fragile geriatric populations.
- With a structured mentorship programme, a regional hospital can safely transition to a high-volume pacing centre, achieving an expert-level safety plateau (3.4% major complication rate) after 100 cases—a safety profile non-inferior to established tertiary standards.
- Decentralised, high-volume pacing programmes provide a scalable, evidence-based alternative to strict centralisation, effectively eliminating access inequality and patient travel burdens without compromising clinical outcomes.

---

3. Introduction

The burden of symptomatic bradyarrhythmias is increasing across Europe, driven primarily by an ageing population. While the European Society of Cardiology (ESC) provides clear clinical guidelines for the management of these conditions [1], the equitable implementation of these services in rural and regional areas remains a profound logistical and policy challenge.

Current healthcare paradigms strongly favour the centralisation of device implantations in tertiary, university-affiliated hospitals [8]. This model is predicated on the assumption that high procedural volumes in centralised locations are the sole means of ensuring patient safety and minimising complications [2]. However, for geriatric populations living in remote regions, this creates a severe "geographical barrier" [9]. Forced centralisation often leads to delayed treatments, logistical hardships for patients and their families, and substantial psychological stress, highlighting a critical gap in healthcare equity.

We hypothesised that a regional hospital could bypass the limitations of strict centralisation and provide "Centre of Excellence" quality care locally. By implementing a structured mentorship programme with a national tertiary centre (Pauls Stradins Clinical University Hospital, Latvian Cardiology Centre), we aimed to evaluate the clinical feasibility, safety, and learning curve associated with establishing a high-volume cardiac pacing programme in a regional geriatric population.

In post-Soviet healthcare systems, such as in Latvia, cardiac care has traditionally followed a highly centralized model. This legacy often forces geriatric patients from remote regions to travel long distances to the capital for life-saving procedures, creating significant "distance decay" in healthcare quality and accessibility. There is a critical

need to evaluate whether transitioning to a decentralized, regionalized model can maintain surgical safety while improving patient outcomes.

---

4. Methods

Study design and participants

This was a prospective cohort study conducted at the Regional General Hospital (Daugavpils, Latvia) over 2 years (2024–2025). Daugavpils Regional Hospital, the primary multi-profile medical facility serving the Latgale region in southeastern Latvia. To address geographical health inequities, we implemented a decentralized Hub-and-Spoke healthcare delivery model. In this framework, our regional hospital (the ‘Spoke’) was developed into a high-volume pacing center serving a regional catchment area of approximately 250,000 inhabitants, many of whom reside up to 250 km from the national tertiary ‘Hub’ in the capital. This regionalization strategy was supported by a formal mentorship program from the national center, aimed at transitioning from a single-center monopoly to a distributed network of specialized care without compromising the gold standard of surgical safety. We enrolled 173 consecutive patients who presented with symptomatic bradycardia meeting standard ESC criteria for permanent pacing [1]. There were no clinical exclusion criteria, ensuring the cohort represented a true "real-world" demographic. The study protocol was approved by the Institutional Ethics Committee, and all participants provided written informed consent prior to the procedures. The research was conducted in full accordance with the Declaration of Helsinki.

Procedures and Mentorship Programme

All implantations were performed using a standardised surgical protocol via subclavian vein puncture. Depending on the clinical indication, patients received either dual-chamber (DDD/DDDR) or single-chamber (VVI/VVIR) systems.

Crucially, the programme was structured around a mentorship-based transition model. Initial cases were performed under the direct proctorship of senior electrophysiologists from the Latvian Cardiology Centre. As the regional team's competence developed, the proctorship transitioned to remote case review and complex case consultation, guiding the regional operators from the initial "acquisition phase" through to "expertise."

Outcomes

The **primary endpoint** was clinical safety, defined as the incidence of 30-day major procedure-related complications (including lead dislocation, systemic infection, pneumothorax, or procedure-related mortality). **Secondary endpoints** included procedural efficiency (measured by total procedure time and fluoroscopy time) and changes in patient-reported quality of life (QoL). QoL and symptom burden were assessed pre-operatively and at 6 months post-operatively using the validated AQUAREL (pacemaker-specific) [4] and SF-36 (general health) [5] questionnaires. Subjective patient satisfaction and trust were assessed using a 10-point Visual Analogue Scale (VAS).

Statistical analysis

To evaluate the evolution of procedural safety and efficiency, a Cumulative Sum (CUSUM) analysis was employed to map the surgical learning curve across the 173 consecutive procedures [6]. Continuous variables were expressed as mean ± standard deviation (SD). Pre- and post-operative QoL scores were compared using a paired Student's t-test. P -value of was considered statistically significant. All statistical analyses were performed using IBM SPSS Statistics for Windows, Version 29.0 (IBM Corp., Armonk, NY, USA).

---

5. Results

Baseline Clinical and Procedural Characteristics

The baseline demographic, clinical, and intraoperative characteristics of the study cohort are summarised in **Table 1**. The study population reflects a typical real-world geriatric demographic, with a mean age of 78.1 ± 9.2 years (range 48–97 years) and a slight female predominance (54.3%). The primary clinical indications for permanent pacemaker implantation were high-grade atrioventricular (AV) block, which accounted for 61.8% of cases, followed by sick sinus syndrome (SSS) in 38.2%.

The vast majority of the cohort (85.5%) underwent first-time (de novo) device implantation, while 14.5% underwent elective generator replacement. Dual-chamber pacing systems (DDD/DDDR) were implanted in 66.5% of patients, whereas 33.5% received single-chamber (VVI/VVIR) devices. Intraoperative testing demonstrated excellent electrical stability and optimal lead performance across all procedures (Table 1).

Procedural Efficiency and Learning Curve

Analysis of procedural times revealed a distinct three-phase evolution (**Figure 1**):

1. **Acquisition (Cases 1–57):** Mean procedure time was 120 minutes.
2. **Stabilisation (Cases 58–115):** Mean procedure time decreased to 85 minutes.
3. **Expertise (Cases 116–173):** Mean procedure time plateaued at 78 minutes.

By the final phase, the regional programme achieved high technical efficiency and radiation safety, characterised by a mean total procedure time of 78.4 ± 14.2 minutes and an exceptionally low mean fluoroscopy exposure time of 3.9 ± 1.1 minutes.

Safety and Complications (CUSUM Analysis)

The overall total complication rate across the entire cohort was 5.7%. Lead dislocations occurred in 2.3% of cases, predominantly within the first 60 cases. There was no procedure-related mortality or systemic infection.

The CUSUM Safety Learning Curve (**Figure 2**) demonstrated a significant shift across the three phases. In Phase 1 (Acquisition), the complication rate was 8.7%, primarily associated with prolonged venous access and early technical adjustments. In Phase 2 (Stabilisation), safety improved markedly, with complications dropping to 5.2% as surgical routines standardised. In Phase 3 (Expert Plateau), the complication rate reached an expert-level plateau of 3.4%. Complications in this final phase were strictly

associated with complex patient anatomy (e.g., severely dilated right atria) rather than technical errors.

Patient-Reported Outcomes and Quality of Life

Patient-reported outcomes and health-related QoL at the 6-month follow-up demonstrated highly significant improvements across all measured metrics (**Table 2**).

Evaluation of pacemaker-specific symptom burden (AQUAREL) revealed a marked reduction in clinical symptoms. Mean scores for chest pain and discomfort decreased from 42.5 ± 12.1 at baseline to 18.2 ± 6.4 at 6 months. Patients also reported significant relief from arrhythmia symptoms and a pronounced reduction in exertional dyspnoea. General QoL (SF-36) showed corresponding enhancements, with Physical Functioning, Vitality, and Mental Health domains all improving significantly.

Subjective patient satisfaction (VAS) was exceptionally high at 6 months. Patients reported an overall satisfaction score for the procedure of 9.9 ± 0.2. The cohort demonstrated profound trust in the regional medical team (9.8 ± 0.4) and high satisfaction regarding the ease of access and proximity to care (9.7 ± 0.5).

---

6. Discussion

Summary of main findings

Our study demonstrates that establishing a high-volume regional pacing programme is not only clinically feasible but highly safe. Through a structured mentorship approach, our regional centre successfully navigated the surgical learning curve, reducing initial complication rates from 8.7% to a stabilised expert plateau of just 3.4% after roughly 100 cases. Concurrently, the programme delivered profound improvements in patient quality of life and generated immense trust within the local community.

Our findings dismantle the "centralization myth" by proving that high-volume regional centers can achieve safety plateaus (3.4% major complication rate) non-inferior to international benchmarks. The implementation of the Hub-and-Spoke model effectively eliminated geographical barriers for a fragile geriatric population. The significant improvement in patient-reported quality of life (AQUAREL and SF-36 scores) and the high level of trust (9.8/10) demonstrate that local accessibility is a key determinant of success in modern cardiac rhythm management.

Comparison with other studies

The data decisively challenge the "low-volume, high-risk" trap often associated with regional hospitals. By concentrating local pathology, we achieved an expertise phase complication rate (3.4%) that is significantly lower than the EHRA average of 7.8% reported by larger, established centres [3, 8]. Furthermore, our stabilised mean fluoroscopy time of 3.9 minutes places our regional centre in the highest-performing quartile of Western European benchmarks, underscoring strict adherence to radiation safety protocols.

Clinical and Policy Implications

For an ageing demographic (mean age 78.1 in our cohort), the logistical and psychological burden of travelling to a distant tertiary centre is substantial [9]. The exceptionally high trust score (9.8/10) reported in our study strongly suggests that receiving advanced procedural care "close to home" by a familiar medical team significantly reduces perioperative anxiety. Furthermore, the inclusion of 14.5% elective replacements indicates that our regional centre has matured into a full-cycle facility, capable of safely managing long-term device replacements [7]. This decentralised model offers a sustainable solution to regional health disparities.

Strengths and Limitations

The primary strength of this study is its prospective, consecutive "real-world" design, which eliminates selection bias. The use of a transparent CUSUM analysis provides a mathematically rigorous mapping of the surgical learning curve [6], offering a precise benchmark (100 cases) for other regional hospitals aiming to adopt this model. Limitations include the single-centre nature of the study and a cohort size that, while substantial for a regional Baltic centre, is relatively small compared to multinational registries. Additionally, the study lacks a direct, randomised control group within the same institution; however, this was mitigated by benchmarking our outcomes against established EHRA registry data [3].

Conclusion

Establishing a high-volume regional pacing programme is a safe, efficient, and highly necessary strategy for modern European cardiology. Our findings dismantle the traditional necessity of strict centralisation, proving that with appropriate mentorship, regional centres can safely eliminate geographical barriers, achieve outcomes non-inferior to Western European tertiary standards, and foster unparalleled patient trust.

---

7. Declarations

Contributors SP conceptualised the study and designed the prospective protocol. **SP** , **KK** and **NN** performed the clinical procedures and were responsible for procedural safety and clinical supervision. **DV** were responsible for data collection, patient follow-up, and the administration of quality-of-life questionnaires. **DV** performed the statistical and CUSUM analyses. **SP** drafted the initial manuscript. **KK, NN** and **OK** provided expert mentorship and critically revised the manuscript for important intellectual content. All authors had full access to all the data in the study and accept responsibility for the decision to submit for publication

Data sharing statement: De-identified individual participant data, along with the study protocol and statistical analysis plan, will be made available upon reasonable request to the corresponding author, following publication, for researchers whose proposed use of the data has been approved by an independent review committee.

Declaration of interests: The authors declare no competing or financial interests.

Acknowledgements We extend our deepest gratitude to the electrophysiology team at the Latvian Cardiology Centre, Pauls Stradins Clinical University Hospital, for their

invaluable mentorship, proctorship, and support during the acquisition phase of this regional programme.

Funding None.

8. Declaration of generative AI and AI-assisted technologies in the writing process

During the preparation of this work the authors used Google Gemini and NotebookLM in order to translate the manuscript into academic British English, structure the text according to the journal's guidelines, and format the clinical data descriptions. After using these tools, the authors reviewed and edited the content as needed and take full responsibility for the final content of the publication.

9. References

[1] Glikson M, Nielsen JC, Kronborg MB, Michowitz Y, Auricchio A, Barbash IM, et al. 2021 ESC Guidelines on cardiac pacing and cardiac resynchronization therapy: Developed by the Task Force on cardiac pacing and cardiac resynchronization therapy of the European Society of Cardiology (ESC) With the special contribution of the European Heart Rhythm Association (EHRA). *Eur Heart J*. 2021;42(35):3427-3520.

[2] Udo EO, Zuithoff NP, van Hemel NM, de Cock CC, Hendriks T, Doevendans PA, et al. Incidence and predictors of short- and long-term complications in pacemaker therapy: the FOLLOWPACE study. *Heart*. 2012;98(9):728-732.

[3] Arribas F, Auricchio A, Boriani G, Brugada J, Deharo JC, Hindricks G, et al. Statistics on the use of cardiac electronic devices and electrophysiological procedures in the European Society of Cardiology countries: 2014 report from the European Heart Rhythm Association (EHRA). *Europace*. 2015;17(suppl 1):i1-i75.

[4] Stofmeel PAM, Postema S, Kelder JC, Grobbee DE, van Hemel NM. The AQUAREL, a disease-specific quality of life questionnaire for pacemaker patients. *J Clin Epidemiol*. 2001;54(5):512-519.

[5] Ware JE Jr, Sherbourne CD. The MOS 36-item short-form health survey (SF-36). I. Conceptual framework and item selection. *Med Care*. 1992;30(6):473-483.

[6] Biau DJ, Porcher R, Ingrand P. The cumulative sum (CUSUM) control chart for monitoring outcomes in healthcare. *Qual Saf Health Care*. 2008;17(3):233-238.

[7] Krahn AD, Lee DS, Birnie D, Healey JS, Crystal E, Dorian P, et al. Predictors of short-term complications after implantable cardioverter-defibrillator replacement: results from the Ontario ICD Database. *Circ Arrhythm Electrophysiol*. 2011;4(2):136-142.

[8] Proclemer A, Zecchin M, D'Onofrio A, Botto GL, Facchini M, Rebellato L, et al. The Pacemaker and Implantable Cardioverter-Defibrillator Registry of the Italian Association of Arrhythmology and Cardiac Pacing—Annual Assessment 2016. *G Ital Cardiol (Rome)*. 2018;19(2):119-131.

[9] Rittenhouse BE, O'Brien PE. Geographical barriers to access and healthcare outcomes: A systematic review of cardiovascular care in rural populations. *Health Policy*. 2019;123(11):1084-1092.

---

10. Tables and Figures

- **Figure 1:** Learning Curve (Time vs. Case Number).
- **Figure 2:** CUSUM Safety Curve (Complications vs. Case Number).
- **Figure 3:** Benchmarking Bar Chart (Our Centre vs. EHRA Averages).

10.1. Figure Legends

Figure 1: Evolution of procedural efficiency across the surgical learning curve. (see in Supplementary Material).The graph demonstrates the relationship between cumulative case volume (x-axis) and total procedure time in minutes (y-axis). The overall learning curve is demarcated into three distinct clinical phases: the initial Acquisition phase (Cases 1–57; mean time 120 min), the Stabilisation phase (Cases 58–115; mean time 85 min), and the final Expertise phase (Cases 116–173; mean time 78 min). The trendline illustrates a continuous reduction in procedural duration, highlighting that optimal surgical efficiency is consistently achieved and stabilised after approximately 100 cases.

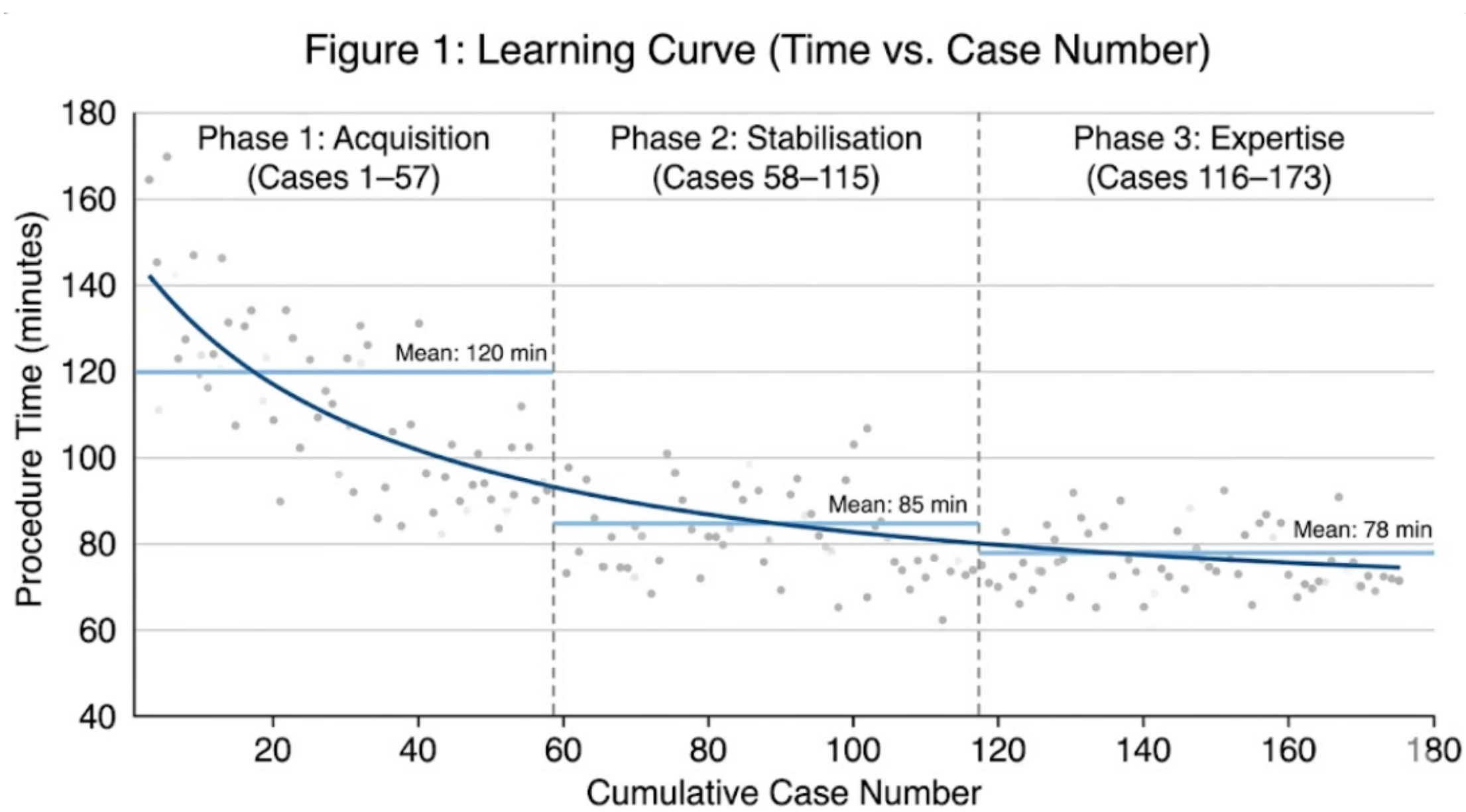

Figure 2: Cumulative Sum (CUSUM) safety analysis of major procedure-related complications.

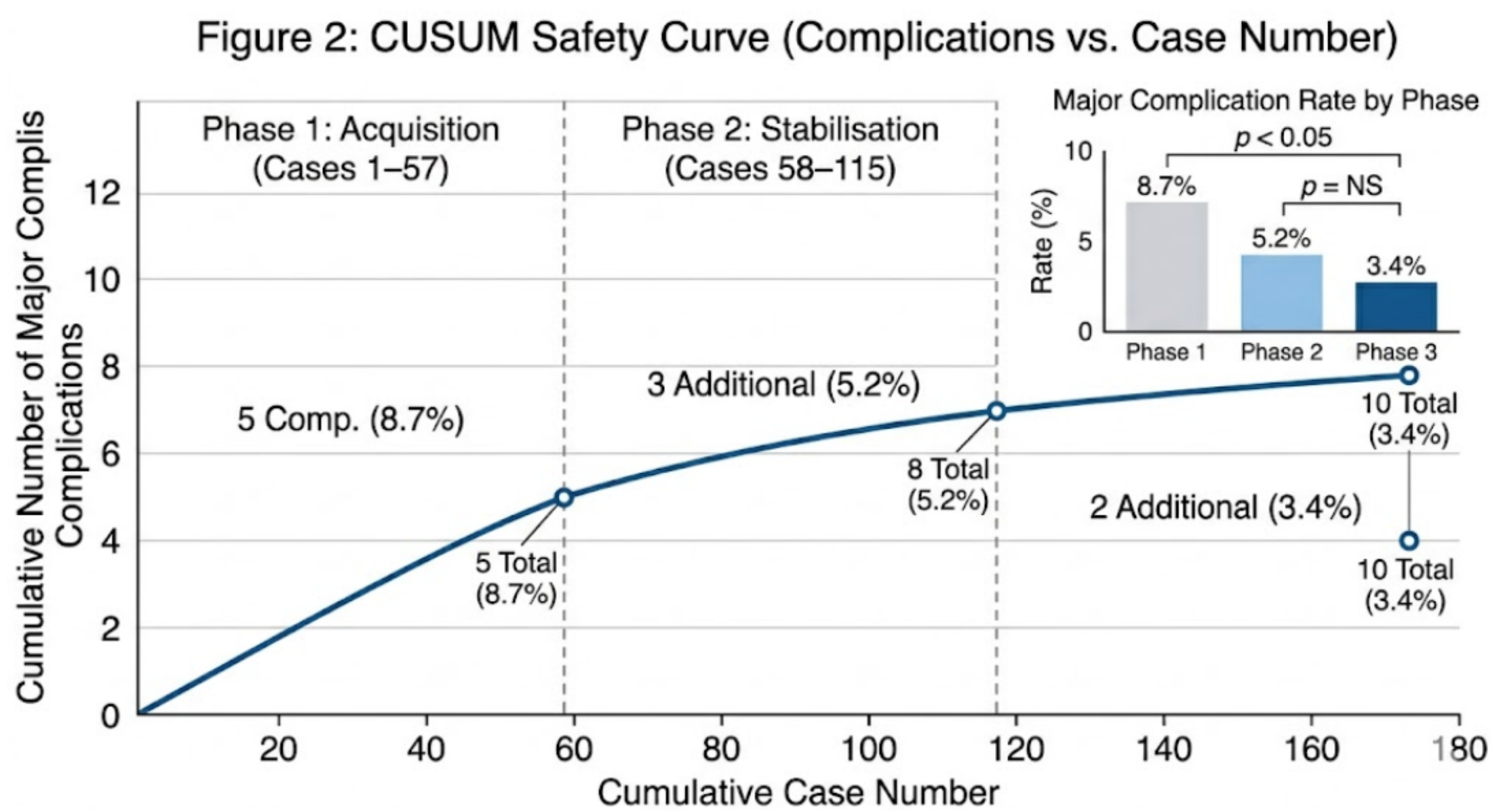

This CUSUM curve tracks the cumulative incidence of major complications across the 173 consecutive pacemaker implantations. The slope of the curve represents the complication rate at a given stage of the programme's maturity. A steeper slope is observed during Phase 1 (Acquisition, 8.7% rate), reflecting early procedural adjustments. The curve visibly flattens through Phase 2 (Stabilisation, 5.2% rate) and reaches a sustained safety plateau in Phase 3 (Expertise), where the major complication rate drops to 3.4%. The embedded bar chart highlights the statistically significant reduction in major complications between the initial and expert phases ($p < 0.05$).

Figure 3: Benchmarking of clinical safety and procedural outcomes against European Heart Rhythm Association (EHRA) averages.

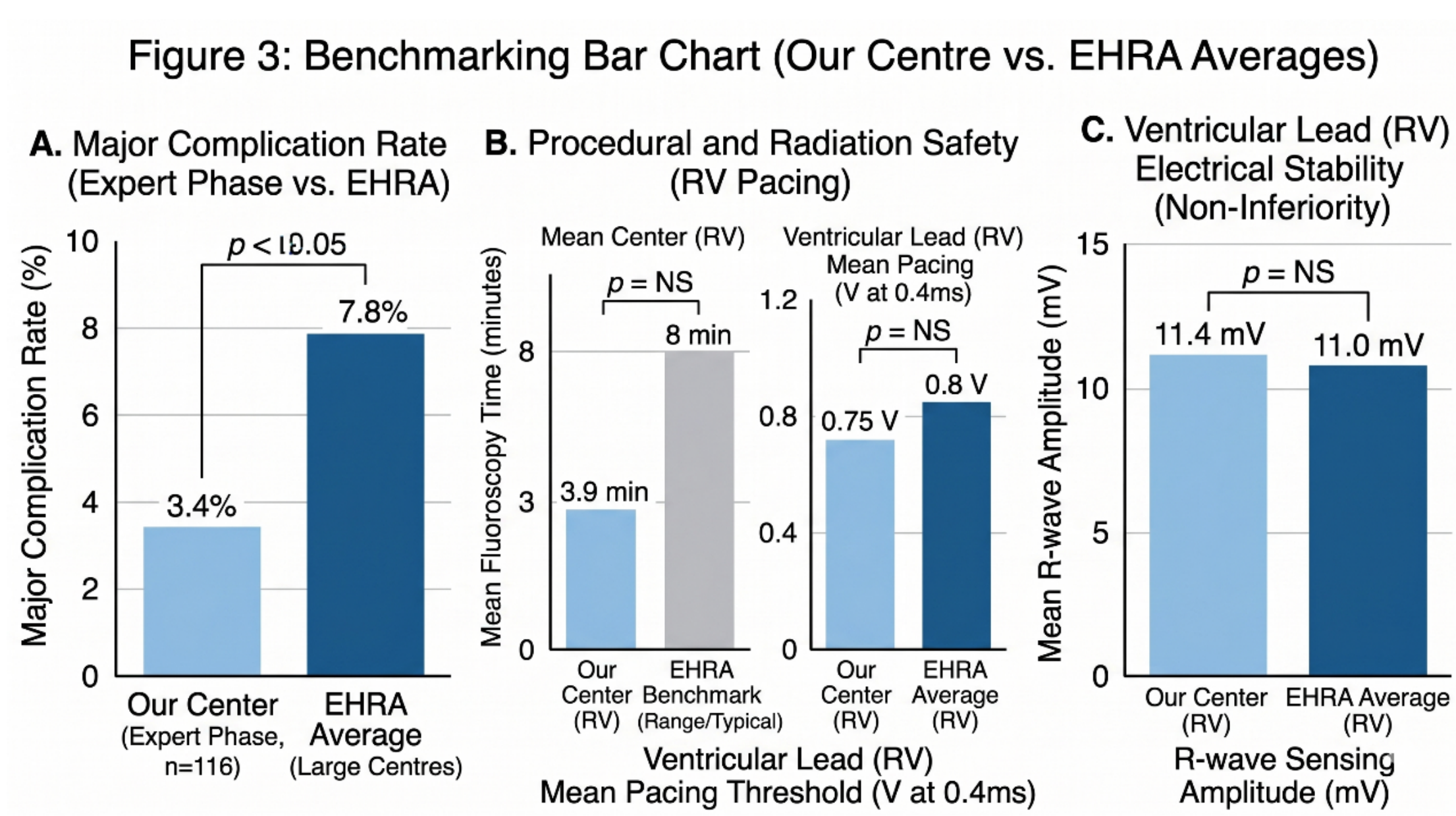

(A) The major complication rate of the regional centre during its mature

Expertise Phase (n=116) compared to the established EHRA registry average. The regional programme demonstrated a significantly lower complication rate (3.4% vs. 7.8%; $p < 0.05$).

(B) Procedural and radiation safety metrics, illustrating the centre's exceptionally low mean fluoroscopy time (3.9 minutes) compared to typical Western European benchmarks.

(C) Intraoperative electrical stability (mean R-wave sensing amplitude and ventricular pacing threshold), confirming that the regional centre achieves optimal, non-inferior lead performance parameters.

10.2. Tables

- **Table 1:** Baseline Clinical Characteristics and Intraoperative Electrical Parameters of the Study Cohort (n=173). *(Refer to previous submission)*
- **Table 2:** Patient-Reported Outcomes and Quality of Life Assessment (n=173). *(Refer to previous submission)*

Table 1. Baseline Clinical Characteristics and Intraoperative Electrical Parameters of the Study Cohort (n=173)

Characteristic	Value (Mean ± SD or n, %)
**Demographics**	
Age, years (Mean ± SD)	78.1 ± 9.2
Age range, years	48 – 97
Gender (Female/Male)	94 (54.3%) / 79 (45.7%)
**Clinical Indications**	
AV Block (II-III degree)	107 (61.8%)
Sick Sinus Syndrome (SSS)	66 (38.2%)
**Procedure Details**	
Dual-Chamber (DDD/DDDR)	115 (66.5%)
Single-Chamber (VVI/VVIR)	58 (33.5%)
Elective Device Replacement	25 (14.5%)
First-time Implantation	148 (85.5%)

Characteristic	Value (Mean ± SD or n, %)
**Intraoperative Electrical Parameters**	
**Ventricular Lead (RV)**	
**— R-wave Sensing (mV)**	**11.4 ± 3.8**
**— Pacing Threshold (V at 0.4ms)**	**0.75 ± 0.21**
**— Impedance (Ω)**	**548 ± 112**
**Atrial Lead (RA) — *DDD cases only***	
**— P-wave Sensing (mV)**	**2.8 ± 1.1**
**— Pacing Threshold (V at 0.4ms)**	**0.82 ± 0.28**
**— Impedance (Ω)**	**512 ± 95**
**Efficiency Metrics**	
**Mean Procedure Time (min)**	**78.4 ± 14.2**
**Mean Fluoroscopy Time (min)**	**3.9 ± 1.1**

Table 2. Patient-Reported Outcomes and Quality of Life Assessment (n=173)

Domain / Metric	Pre-Operative (Baseline)	Post-Operative (6 Months)	p-value
**AQUAREL Score (Pacemaker-specific)**			
— Chest Pain / Discomfort	42.5 ± 12.1	**18.2 ± 6.4**	< 0.001
— Arrhythmia Symptoms	58.1 ± 15.4	**12.5 ± 5.2**	< 0.001
— Exertional Dyspnea (Shortness of breath)	64.3 ± 18.2	**24.1 ± 8.7**	< 0.001
**SF-36 Health Survey Domains**			
— Physical Functioning	38.4 ± 10.5	**62.7 ± 12.8**	< 0.001
— Vitality / Energy Levels	31.2 ± 9.8	**58.4 ± 11.2**	< 0.001
— Mental Health / Emotional Well-being	45.6 ± 11.4	**71.2 ± 10.5**	< 0.001
**Subjective Satisfaction (VAS 1-10)**			

Domain / Metric	Pre-Operative (Baseline)	Post-Operative (6 Months)	p-value
— Trust in Regional Medical Team	N/A	**9.8 ± 0.4**	—
— Ease of Access (Proximity to home)	N/A	**9.7 ± 0.5**	—
— Overall Procedure Satisfaction	N/A	**9.9 ± 0.2**	—

1

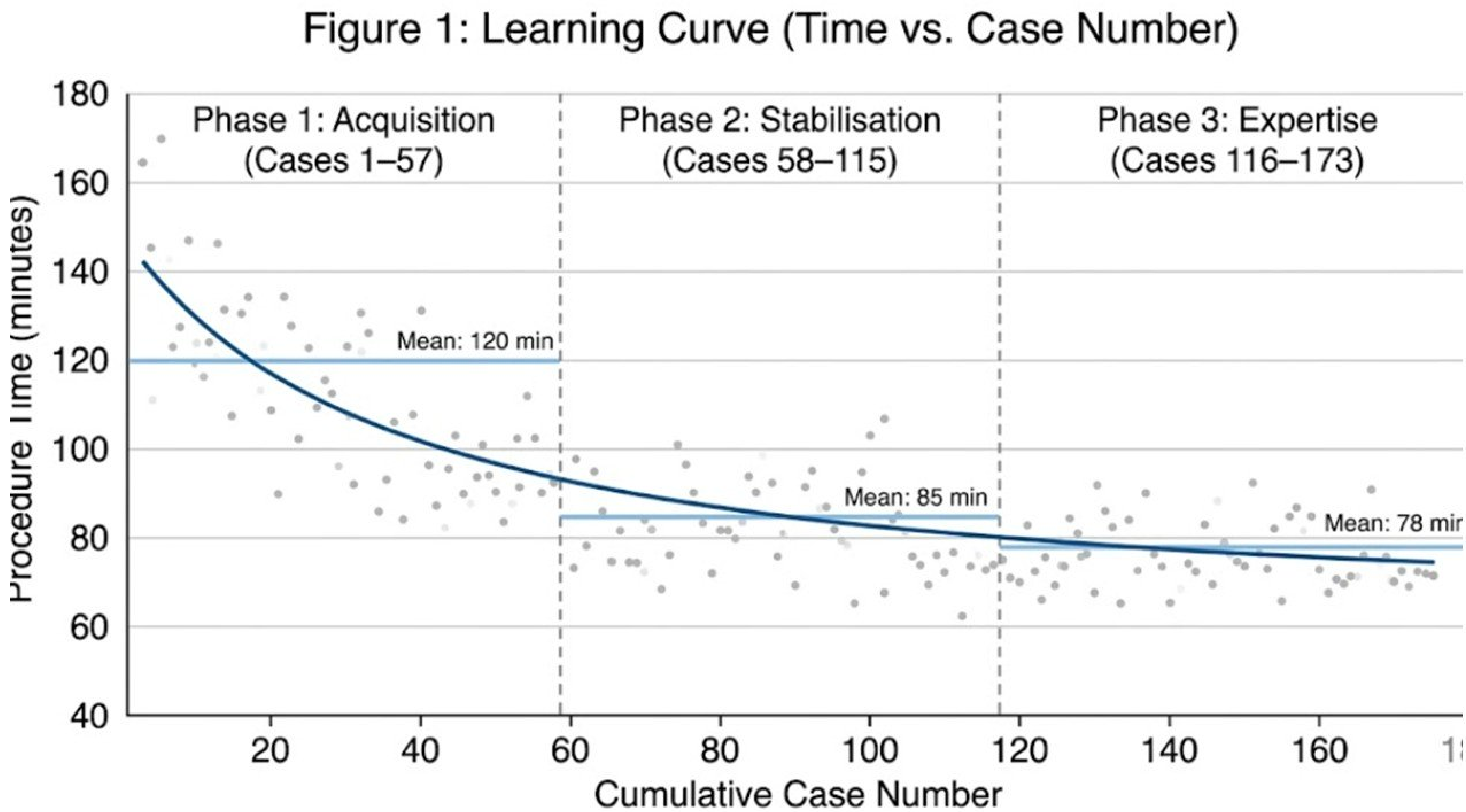
Figure 1: Learning Curve (Time vs. Case Number)
Phase 1: Acquisition (Cases 1–57)
Phase 2: Stabilisation (Cases 58–115)
Phase 3: Expertise (Cases 116–173)
Mean: 120 min
Mean: 85 min
Mean: 78 mir
Procedure Time (minutes)
Cumulative Case Number
180
160
140
120
100
80
60
40
20
40
60
80
100
120
140
160

Figure 2: CUSUM Safety Curve (Complications vs. Case Number)

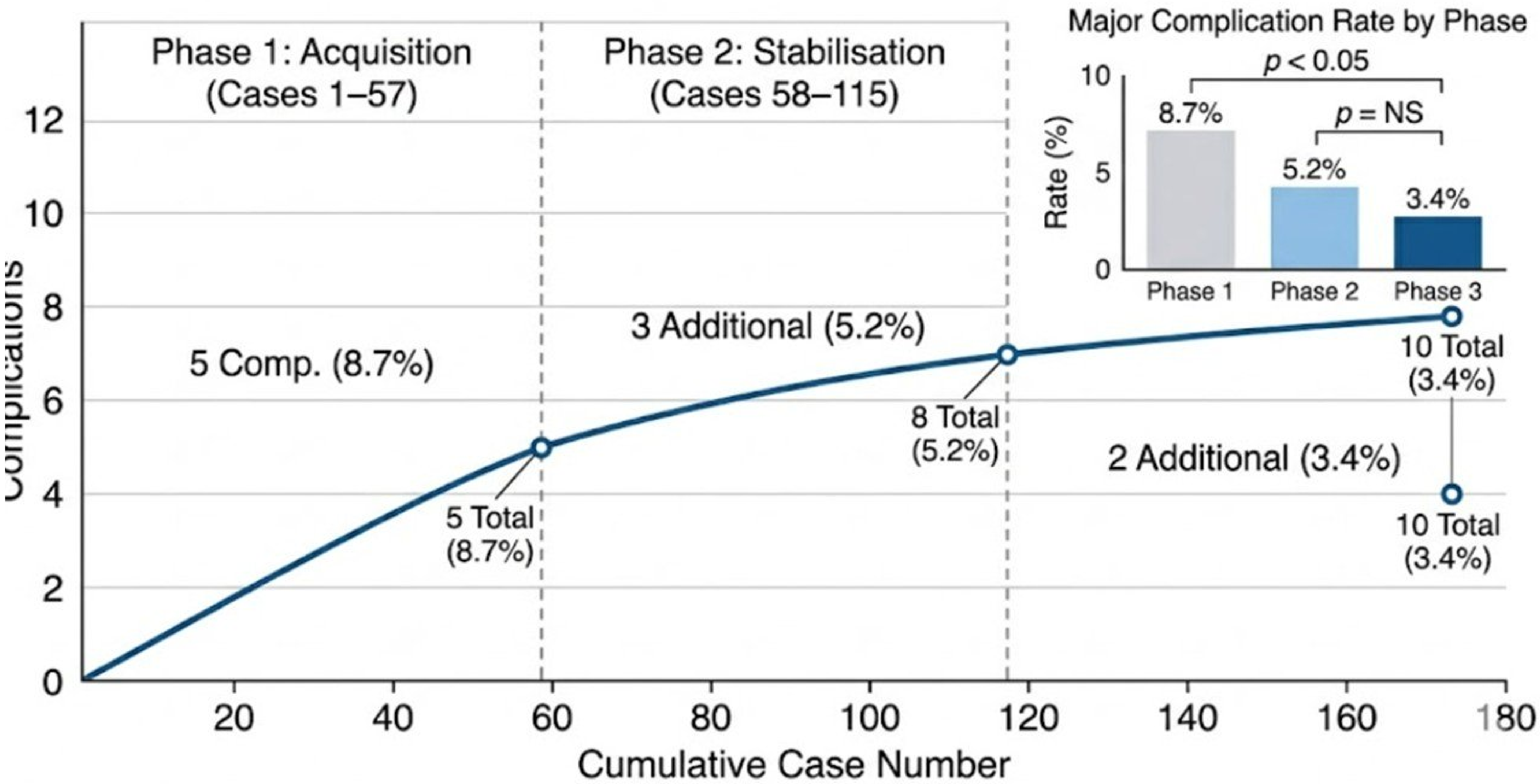

Figure 3: Benchmarking Bar Chart (Our Centre vs. EHRA Averages)

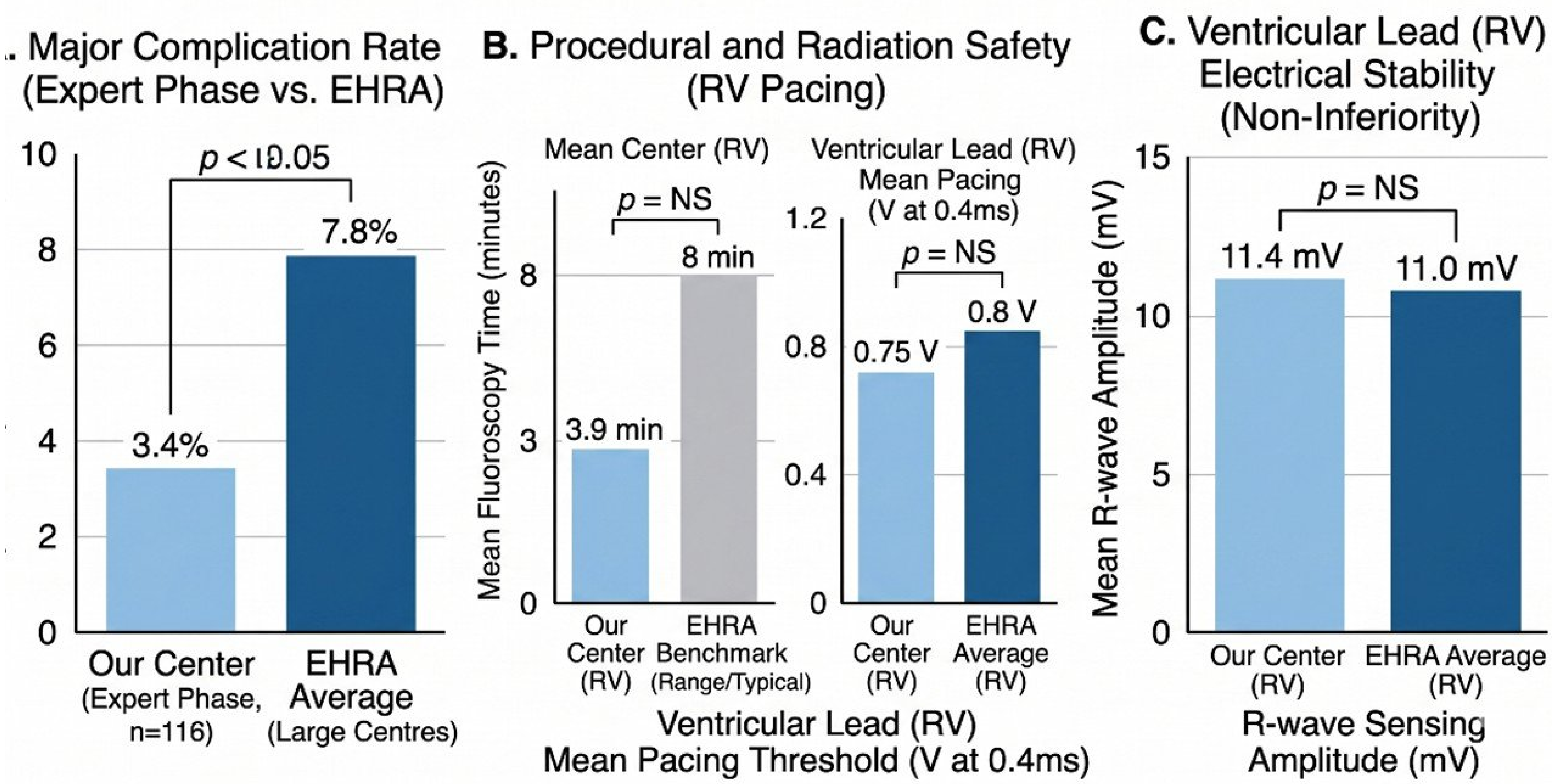

Table 1. Baseline Clinical Characteristics and Intraoperative Electrical Parameters of the Study Cohort (n=173)

Characteristic	Value (Mean ± SD or n, %)
Demographics	
Age, years (Mean ± SD)	78.1 ± 9.2
Age range, years	48 – 97
Gender (Female/Male)	94 (54.3%) / 79 (45.7%)
Clinical Indications	
AV Block (II-III degree)	107 (61.8%)
Sick Sinus Syndrome (SSS)	66 (38.2%)
Procedure Details	
Dual-Chamber (DDD/DDDR)	115 (66.5%)
Single-Chamber (VVI/VVIR)	58 (33.5%)
Elective Device Replacement	25 (14.5%)
First-time Implantation	148 (85.5%)
Intraoperative Electrical Parameters	
Ventricular Lead (RV)	
— R-wave Sensing (mV)	11.4 ± 3.8
— Pacing Threshold (V at 0.4ms)	0.75 ± 0.21
— Impedance (Ω)	548 ± 112
Atrial Lead (RA) — *DDD cases only*	
— P-wave Sensing (mV)	2.8 ± 1.1
— Pacing Threshold (V at 0.4ms)	0.82 ± 0.28
— Impedance (Ω)	512 ± 95
Efficiency Metrics	
Mean Procedure Time (min)	78.4 ± 14.2

Characteristic	Value (Mean ± SD or n, %)
Mean Fluoroscopy Time (min)	3.9 ± 1.1

Table 2. Patient-Reported Outcomes and Quality of Life Assessment (n=173)

**Domain / Metric**	**Pre-Operative (Baseline)**	**Post-Operative (6 Months)**	**p-value**
**AQUAREL Score (Pacemaker-specific)**			
— Chest Pain / Discomfort	42.5 ± 12.1	**18.2 ± 6.4**	< 0.001
— Arrhythmia Symptoms	58.1 ± 15.4	**12.5 ± 5.2**	< 0.001
— Exertional Dyspnea (Shortness of breath)	64.3 ± 18.2	**24.1 ± 8.7**	< 0.001
**SF-36 Health Survey Domains**			
— Physical Functioning	38.4 ± 10.5	**62.7 ± 12.8**	< 0.001
— Vitality / Energy Levels	31.2 ± 9.8	**58.4 ± 11.2**	< 0.001
— Mental Health / Emotional Well-being	45.6 ± 11.4	**71.2 ± 10.5**	< 0.001
**Subjective Satisfaction (VAS 1-10)**			
— Trust in Regional Medical Team	N/A	**9.8 ± 0.4**	—
— Ease of Access (Proximity to home)	N/A	**9.7 ± 0.5**	—
— Overall Procedure Satisfaction	N/A	**9.9 ± 0.2**	—

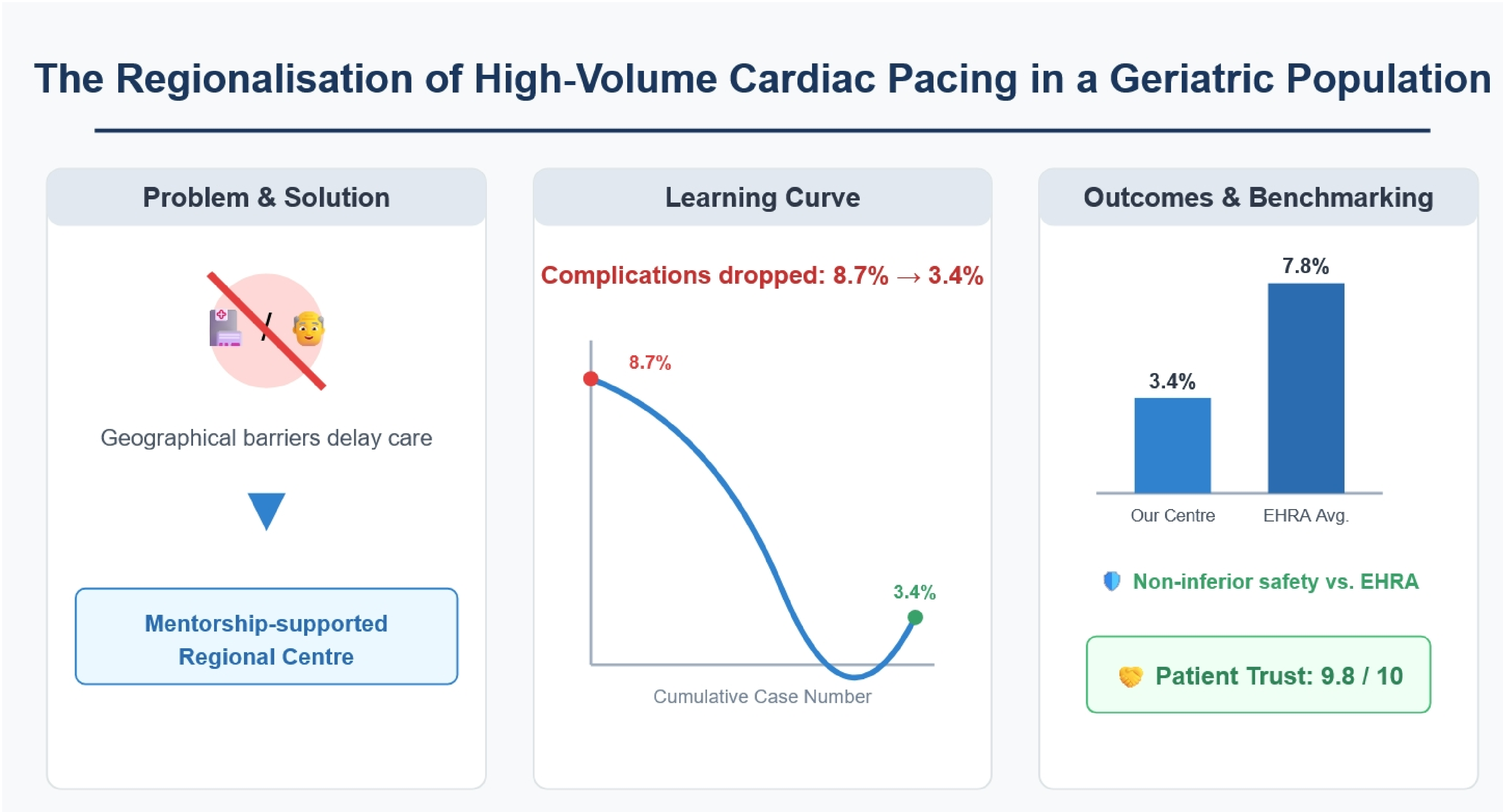
The Regionalisation of High-Volume Cardiac Pacing in a Geriatric Population
Problem & Solution
Geographical barriers delay care
Mentorship-supported Regional Centre
Learning Curve
Complications dropped: 8.7% → 3.4%
8.7%
3.4%
Cumulative Case Number
Outcomes & Benchmarking
7.8%
3.4%
Our Centre
EHRA Avg.
Non-inferior safety vs. EHRA
Patient Trust: 9.8 / 10
